# Gene Polymorphisms Associated with Central Precocious Puberty and Hormone Levels in Chinese Girls

**DOI:** 10.1155/2022/9450663

**Published:** 2022-08-21

**Authors:** Yunwei Li, Na Tao, Minghui Chen, Jiang Chu, Xinwei Huang, Xiangyang Kong

**Affiliations:** ^1^Faculty of Life Science and Biotechnology, Kunming University of Science and Technology, Kunming 650500, Yunnan, China; ^2^Medical School, Kunming University of Science and Technology, Kunming 650500, Yunnan, China; ^3^Department of Pharmacy, Kunming Children's Hospital, Kunming 650228, Yunnan, China; ^4^Department of Endocrinology, Genetics and Metabolism of Children, Kunming Children's Hospital, Kunming 650228, Yunnan, China; ^5^Translational Research Institute of Brain and Brain-Like Intelligence, Shanghai Fourth People's Hospital, School of Medicine, Tongji University, Shanghai 200434, China

## Abstract

Central precocious puberty (CPP) is associated with adverse health outcomes in females; however, CPP pathogenesis remains unclear. In this study, we investigated the association of 20 single nucleotide polymorphisms (SNPs) in eight genes with CPP risk and hormone levels. A case-control study on 247 and 243 girls with and without CPP, respectively, was conducted at Kunming Children's Hospital, China, from September 2019 to August 2020. The genotype of the SNPs and their haplotypes were identified. Additionally, the effects of the polymorphisms on hormone levels were investigated. Three variants (rs10159082, rs7538038, and rs5780218) in *KISS1* and two variants (rs7895833 and rs3758391) in *SIRT1* were related to an increased CPP risk (odds ratio (OR) = 1.524, 1.507, 1.409, 1.348, and 1.737; 95% confidence interval (CI) = 1.176–1.974, 1.152–1.970, 1.089–1.824, 1.023–1.777, and 1.242–2.430, respectively). Rs3740051in *SIRT1* and rs1544410 in *VDR* reduced CPP risk (OR = 0.689, 0.464; 95% CI, 0.511–0.928, 0.232–0.925, respectively). Rs1544410, rs7975232, and rs731236 in *VDR* were negatively correlated with peak follicle-stimulating hormone (FSH; *β* = −2.181; *P*=0.045), basal FSH (*β* = −0.391; *P*=0.010), and insulin-like growth factor (*β* = −50.360; *P*=0.041) levels, respectively. *KISS1*, *SIRT1*, and *VDR* variants were associated with CPP susceptibility, and *VDR* SNPs influenced hormonal levels in Chinese females with CPP. In particular, *VDR* polymorphism rs1544410 was associated with both CPP risk and GnRH-stimulated peak FSH levels. Further functional research and large-scale genetic studies of these loci and genes are required to confirm our findings.

## 1. Introduction

Precocious puberty is a global phenomenon that predominantly occurs in females; it is defined as puberty that starts before the age of nine in males and before the age of eight in females [1]. In the United States, breast development (Tanner stage ≥(2) is observed in 18.3%, 30.9%, and 42.9% of eight-year-old white, Hispanic, and black non-Hispanic females, respectively [2]. In Europe, approximately 5% of females exhibit the onset of breast development before the age of eight [3]. In China, 2.9% of females from six different cities exhibit breast development before the age of eight [4]. However, another recent study from Shanghai primary school in China revealed that 17.2% of females aged 6-7 presented with breast development at Tanner stage ≥2, and 19.0% were diagnosed with precocious puberty [5]. Therefore, precocious puberty has attracted considerable attention owing to its high incidence and adverse effects on health outcomes, including obesity, ischemic heart disease, type 2 diabetes, hypertension, estrogen-dependent cancer [6, 7], and behavioral and mental disorders [8]. Understanding the underlying pathogenesis of this disorder is crucial for effective diagnosis and treatment.

Activation of the hypothalamic–pituitary–gonadal (HPG) axis is a hallmark of pubertal onset. The gonadotropin-releasing hormone (GnRH) secreted from the hypothalamus stimulates the pituitary gland to release gonadotropins such as luteinizing hormone (LH) and follicle-stimulating hormone (FSH). In turn, gonadotropins stimulate gonadal maturation and the synthesis of sex steroids [9]. Premature activation of the HPG axis leads to central precocious puberty (CPP), the most common type of precocious puberty. However, the pathogenesis of CPP is not completely clear. Pubertal onset is modulated by a complex combination of genetic, environmental, nutritional, and socioeconomic factors [10], with genetic factors contributing to 50–80% of the variation in pubertal timing in the general population [11]. Genome-wide association studies have identified hundreds of genetic loci that impact pubertal timing in several ethnic groups, as well as numerous loci variations involved in precocious puberty [12]. Moreover, Vries et al. [13] used segregation analysis and reported that autosomal dominant inheritance with incomplete, sex-dependent penetrance was responsible for familial CPP, which accounts for 27.5% of CPP. Thus, genetic factors may play a major role in the pathogenesis of CPP.

Previous genetic association studies have identified several single nucleotide polymorphisms (SNPs) in KISS1, ER*α*, ER*β*, LIN28B, TMEM38B, and vitamin D receptor (VDR) genes related to precocious puberty or their clinical features [14–22]. In this study, we analyzed the relationship between human CPP risk and carefully selected SNPs according to the following criteria: (a) SNPs previously associated with precocious puberty or their clinical features and (b) SNPs previously associated with metabolic disorders. The SIRT1 gene was selected because it plays an important role in metabolic regulation, which may influence pubertal onset [23]. Moreover, SIRT1 SNPs have been linked with metabolic diseases such as obesity and type 2 diabetes [24–26], and animal studies have demonstrated that the SIRT1 protein advances puberty through the regulation of KISS1 expression, which stimulates GnRH secretion [27]. Thus, we hypothesized that SIRT1 variants may be associated with human CPP risk. Further, the association between the aforementioned SNPs and the hormonal levels in Chinese females with CPP has not been well studied before. Therefore, we conducted a genetic association study to explore the relationships of the selected SNPs with CPP risk and hormonal levels in Chinese females.

## 2. Materials and Methods

### 2.1. Study Subjects

A total of 243 healthy control females and 247 females with CPP were recruited for this study at Kunming Children's Hospital, Kunming City, Yunnan Province, China, from September 2019 to August 2020. According to the 2018 guidelines of the Chinese Medical Association [28], the CPP inclusion criteria were as follows: females exhibiting secondary sex characteristics before the age of eight or menarche before the age of 10; accelerated linear growth and bone age (BA) that exceeds the chronological age (CA) by more than one year; enlarged ovaries and uterus, with at least one ovarian follicle greater than 4 mm in diameter; HPG axis function characterized by peak LH > 3.1–5.0 IU/L and a peak LH/FSH ratio >0.60 according to a GnRH stimulation test. The CPP exclusion criteria were as follows: cases diagnosed with peripheral precocious puberty or precocious puberty caused by an organic lesion, brain tumor, McCune–Albright syndrome, congenital adrenal, hyperplasia, hypothyroidism, etc. Healthy females matched to the age of subjects with CPP were selected as control subjects and exhibited no secondary sexual characteristics, abnormal growth and development, or chronic wasting diseases.

Clinical data including age, height, weight, body mass index (BMI), BA, BA advancement (i.e., BA minus CA), level of estradiol (E2), insulin-like growth factor (IGF-1), basal FSH, basal LH, peak LH, and peak FSH, and the peak LH/FSH ratio were collected and evaluated by a trained pediatric endocrinologist. BA was established from a left-hand radiograph. The GnRH stimulation test was conducted by measuring LH and FSH levels in blood samples taken at 0, 30, 60, and 90 min after intravenous injection of gonadorelin. The levels of LH, FSH, E2, and IGF-1 were detected by chemiluminescence with a Diagnostics Roche cobas e602 analyzer. Informed consent was obtained from parents or guardians of all participants. This study was approved by the ethics committee of Kunming Children's Hospital [No. 20190717003].

### 2.2. DNA Extraction

Ethylenediaminetetraacetic acid anticoagulant-treated whole blood was collected and transferred to a microcentrifuge tube and maintained at −80°C prior to its use for DNA extraction. Purified genomic DNA was extracted from whole-blood leukocytes using the QIAamp DNA blood mini kit (Qiagen, CA, USA) and stored at −20°C before genotyping.

### 2.3. SNP Selection and Genotyping

Twenty SNPs (rs10159082, rs5780218, rs7538038, rs7759938, rs314280, rs364663, rs221634, rs2234693, rs4452860, rs7861820, rs7895833, rs3758391, rs3740051, rs33957861, rs731236, rs7975232, rs1544410, rs2228570, rs1256049, and rs3761170) located in eight genes (KISS1, LIN28B, ER*α*, ER*β*, TMEM38B, SIRT1, VDR, and PLCB1) associated with precocious puberty, its clinical features, or metabolic disorders were screened as the candidate loci according to previous reports [14–22, 24–26]. The minor allele frequency of the selected SNP loci in the Chinese population is more than 0.05 and the polymorphisms do not exhibit complete linkage disequilibrium (LD) (*r*^2^ < 1).

SNPs were genotyped using multiple polymerase chain reaction (PCR) and the Snapshot genotyping method based on the Gene Amp PCR system 96000 (Applied Biosystems, Inc., USA) and ABI 3730XL sequencing platform (Thermo Fisher Scientific, Inc., USA). PCR analysis of the selected SNPs was performed using primers that were prepared using the primer data obtained from NCBI (https://www.ncbi.nlm.nih.gov). Primer details are shown in Table S1.

### 2.4. Statistical Analysis

Clinical data are presented as the mean ± SD or the standard deviation score (SDS). Means of two groups were compared using Student's *t*-test. PLINK version 1.07 was used to perform all genetic analyses [29]. The Hardy–Weinberg equilibrium for each SNP was estimated according to the *χ*^2^ test in the control group. The genotype distribution was then calculated. The genotype of each SNP was assessed using logistic regression analysis with the additive model, and the odds ratio (OR) was calculated with a 95% confidence interval (CI). Linear regression under the additive model was employed to assess the relationships between SNPs and the peak LH, peak FSH, LH/FSH ratio, base LH, base FSH, E2, and IGF-1, adjusting for age and BMI in CPP cases. PLINK and Haploview 4.2 were used to analyze the haplotype blocks of the loci [30], and the pairwise LD was calculated for SNPs within 500 kb. A two-sided *P* value of less than 0.05 was considered statistically significant for all analyses.

## 3. Results

### 3.1. Clinical and Hormonal Parameters of the Study Population

Clinical parameters including age at diagnosis, height, weight, BMI, height SDS, weight SDS, and BMI SDS in the control and CPP groups are presented in Table 1. BA, BA advancement, and hormone parameters—including E2, IGF-1, IGF-1 SDS, basal LH and FSH, peak LH and FSH levels, and peak LH/FSH ratio—of the study population are also shown in Table 1.

### 3.2. Single Nucleotide Polymorphisms Associated with CPP Risk

As summarized in Tables 2, 20 SNP loci were assessed in this study. Genotyping of the selected SNPs had a 100% success rate and the Hardy–Weinberg equilibrium of all SNPs was more than 0.05 in the control samples. To evaluate the correlation between candidate loci in KISS1, LIN28B, ER*α*, ER*β*, TMEM38B, SIRT1, VDR, and PLCB1 genes and CPP risk, the alleles of the selected SNPs were analyzed in both the CPP and the control samples. As shown in Table 3, seven variants in KISS1, SIRT1, and VDR were found to be related to CPP. Three variants (rs10159082, rs7538038, and rs5780218) in KISS1 and two variants (rs7895833 and rs3758391) in SIRT1 showed a significant association with increased CPP risk (OR, 1.524, 1.507, 1.409, 1.348, and 1.737; 95% CI, 1.176–1.974, 1.152–1.970, 1.089–1.824, 1.023–1.777, and 1.242–2.430, respectively). Moreover, rs3740051 in SIRT1 and rs1544410 in VDR were inversely correlated with CPP (OR, 0.689, 0.464; 95% CI, 0.511–0.928, 0.232–0.925, respectively).

### 3.3. Haplotype Analysis in the Study Population

**Figure 1:**
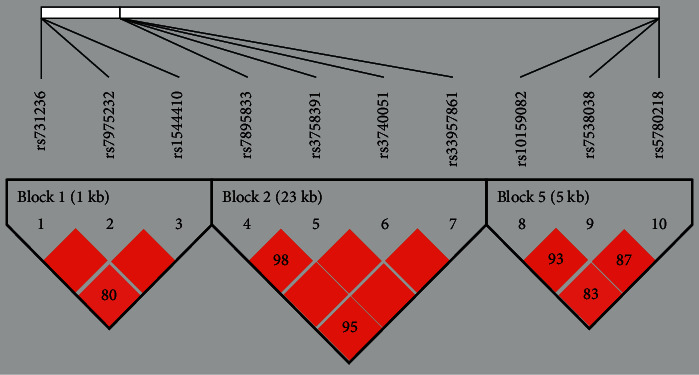
Linkage disequilibrium (LD) plot for VDR, SIRT1, and KISS1 genes. LD coefficient values (D') corresponding to each SNP pair are expressed as a percentage and shown within the respective square. Three SNPs in VDR, four SNPs in SIRT1, and three SNPs in KISS1 comprised one block (Block 1, Block 2, and Block 3, respectively).

The LD coefficient (D') was calculated to represent LD, where a D' value of ≥0.8 indicated that the related SNPs formed one block.  Figure 1 shows the LD plot. Haplotype analysis was performed for SNPs in KISS1, SIRT1, and VDR associated with CPP; the results are shown in Table 4. Haplotype CGdelA in the KISS1 gene was associated with an increased risk of CPP (*P*=0.003), whereas haplotype AAA in KISS1 was associated with a decreased risk of CPP (*P*=0.007). Interestingly, haplotype ACAC in SIRT1 significantly increased CPP risk (*P*=0.0007), whereas haplotype GTGC in SIRT1 reduced CPP risk (*P*=0.014). Haplotype ACAC had a lower *P* value (*P*=0.0007) than that of the individual SNPs (0.0013 < 3*P* < 0.0340), indicating that these SNPs are in LD with a more significantly associated variant; however, we could not confirm whether this difference in *P* values was significant.

### 3.4. Single Nucleotide Polymorphisms Associated with Hormonal Levels

To investigate the effect of hereditary factors on hormonal levels, we performed a linear regression analysis for all SNPs under the additive model, adjusting for age and BMI. As shown in Table 5, rs1544410, rs7975232, and rs731236 in *VDR* were negatively correlated with the peak FSH (*β* = −2.181; *P*=0.045), basal FSH (*β* = −0.391; *P*=0.010), and IGF-1 (*β* = −50.360; *P*=0.041*P* = 0.041) levels.

## 4. Discussion

In the current study, we explored the association of selected SNPs with CPP risk and hormonal levels in Chinese girls. We found seven variations in KISS1, SIRT1, and VDR associated with CPP risk. Three SNPs in VDR showed a significant correlation with peak FSH, basal FSH, and IGF-1 levels in CPP groups. To our knowledge, this is the first study to report an association between SIRT1 polymorphisms and CPP risk in Chinese females. VDR polymorphisms were also shown to affect hormonal levels in Chinese females with CPP for the first time, which gave rise to the possibility that rs1544410 polymorphism in VDR had the protective effect on CPP risk by affecting GnRH-stimulated peak FSH level.

Kisspeptin, encoded by *KISS1*, is regarded as a fundamental gatekeeper of pubertal onset through strong stimulation of the GnRH secretion [31]. For example, the administration of kisspeptin to immature animals induced advanced activation of the HPG axis and puberty development [32]. Moreover, humans with gain-of-function mutations in KISS1, which is expressed on GnRH neurons, showed earlier pubertal onset [33]. These studies imply that the KISS1 gene contributes to the pathogenesis of precocious puberty. In our study, we found that rs10159082, rs7538038, and rs5780218 in KISS1 were significantly associated with an increased risk of CPP. Additionally, our haplotype analysis indicated that haplotypes CGdelA and AAA in KISS1 had a close relationship with the CPP risk. Rs5780218, located in the promoter region of KISS1, may also affect KISS1 transcription [34]. Our results are consistent with the findings reported by Li et al. [14], who showed that rs5780218 plays a role in CPP risk in Chinese females. A large-scale candidate-gene association study also revealed a significant correlation between the rs7538038 variant and the age at menarche [15], and that rs10159082 was in LD with rs7538038 (*r*^2^ = 0.58). These two variants in the KISS1 gene are located in its intron region; however, there is no available literature on their function. Intron variants do not alter the protein sequence of the gene but may impact gene expression or be in LD with another variant that does. Our results revealed that the three variants of KISS1 (rs5780218, rs7538038, and rs10159082) were in LD with each other; accordingly, these associations are not independent. Further functional analysis is required to identify the association between intron variants in KISS1 and CPP risk.

Pubertal timing is driven by not only genetic factors but also nutritional and metabolic factors [23]. Changes in the nutritional or metabolic conditions of an individual can eventually impact the secretion of GnRH [35]. SIRT1, which acts as a key cellular metabolic sensor, can contribute to puberty by controlling the expression of the KISS1 gene [36]. That is, when puberty begins, SIRT1 is separated from the KISS1 promoter, increasing KISS1 transcription. Under conditions of energy excess, such as nutritional excess or early-onset obesity, SIRT1 is prematurely evicted from the promoter of KISS1, which promotes KISS1 expression, causing early puberty [27]. However, few studies have focused on the effect of variants in the SIRT1 gene on CPP risk. Our findings showed that rs3758391 and rs7895833 in SIRT1 could increase CPP risk, whereas rs3740051 in SIRT1 exhibited a protective effect on CPP risk. Moreover, haplotype analysis of these loci indicated that haplotypes GTGC and ACAC in SIRT1 were correlated with the CPP risk. Rs3758391 in SIRT1 is located at the p53-binding site, which is in the distal promoter required for human *SIRT1* transcription. Moreover, the C variation of rs3758391 disrupts the mirror-image symmetry of the p53-binding sequence, affecting *S*IRT1 mRNA expression [37]. Tang et al. [38] reported that rs3758391 variation was significantly related to the occipital cortex SIRT1 expression in humans and that the C allele of rs3758391 was associated with lower SIRT1 expression. In addition, Cruz et al. [26] observed that the minor C allele of rs3758391 conferred a higher risk of type 2 diabetes in the Mexican population. Rs7895833 in *SIRT1* is located 21 kb upstream of *SIRT1*, which may lie within the promoter region, where it could influence *SIRT1* expression [25]. The *SIRT1* rs3740051 variant is also located in the promoter region. Resveratrol, an activator of *SIRT1*, increases energy expenditure in mice [39]. In humans, the *G* allele of SIRT1 rs3740051 is related to higher levels of energy expenditure [39]. These results suggest that the *G* allele at rs3740051 could increase the activity of SIRT1. In addition, *SIRT1* gene polymorphisms rs3758391, rs7895833, and rs3740051 may influence SIRT1 protein activity and expression. Animal studies have reported that decreased *SIRT1* levels are associated with advanced puberty, whereas increased SIRT1 levels are associated with delayed puberty [27]. Considering this study, SIRT1 polymorphisms may affect KISS1 transcription by influencing SIRT1 protein activity and expression, thereby causing susceptibility to CPP.

VDR is a member of the steroid hormone receptor family that serves as a transcriptional activator of many target genes. The four variants rs731236, rs7975232, rs1544410, and rs2228570 in the VDR gene are commonly related to metabolic disorders, such as diabetes, metabolic syndrome (MetS), and polycystic ovarian syndrome (PCOS) [40, 41]. VDR is also crucial for female reproduction [42]. However, literature on the association between VDR polymorphisms and CPP is scarce. Santos et al. [18] reported a relationship between *VDR* polymorphism and precocious pubarche in the studied population. In our study, we found that the mutant allele of rs1544410 in VDR was associated with a reduced risk of CPP. rs1544410, which is located in the 3′-UTR, may be responsible for VDR mRNA stability and expression of VDR [43]. Zhao et al. [40] found rs1544410 to be negatively correlated with the risk of metabolic syndrome in the Northern Chinese population. Sahin et al. [44] showed that the A allele of rs1544410 was associated with a decreased risk of type 1 diabetes. Zhong et al. [45] suggested that the minor A allele of rs1544410 may be a susceptibility marker of diabetic nephropathy in type 2 diabetes in the Chinese population. Collectively, these studies imply that rs1544410 in *VDR* may be a genetic marker for endocrine disorders. This supports our findings on the role of rs1544410 polymorphism in CPP.

Interesting findings emerged from our analysis of the association between the selected SNPs and hormonal parameters. Specifically, we found a negative correlation between VDR rs1544410 polymorphism and peak FSH levels. FSH is essential for female reproductive maturation and, when bound to its *G* protein-coupled receptor (FSHR), regulates folliculogenesis, oocyte selection, and the synthesis of sex steroid hormones in the ovary [46]. With the onset of puberty, FSH stimulates the ovaries to produce the estradiol responsible for breast tissue growth, which is one of the earliest clinical signs of puberty. Hagen et al. [47] found that puberty occurred later in healthy females with the FSHR-29AA (reduced *FSHR* expression) genotype than in individuals with FSHR-29 GG/GA. This strongly suggested that FSH action impacts pubertal timing in females. In addition, VDR mRNA is expressed in the human pituitary gland, and VDR could play a role in controlling the expression of human pituitary hormone genes, including FSH and LH [48]. Taken together, it appears that VDR rs1544410 decreases CPP risk by reducing the GnRH-stimulated peak FSH level. However, further research is required to determine the precise molecular mechanism. Additionally, rs1544410 was negatively correlated with the peak LH level in the CPP groups; however, the correlation was not statistically significant (*P*=0.10). This lack of significance might be attributed to the relatively small number of subjects.

Our study revealed the negative and nonsignificant association between two variants (rs7975232 and rs731236) in the VDR gene and CPP risk; however, we also found a negative and significant correlation between rs7975232 and rs731236 and the level of basal FSH and IGF-1, respectively. The rs7975232 locus, located in the 3′-UTR region, regulates VDR expression by affecting mRNA stability, as does rs731236 [49]. As previously mentioned, VDR may influence FSH secretion. Therefore, rs7975232 polymorphism affects the level of basal FSH. IGF-1, which modulates the expression of the KISS1 gene, plays an important role in pubertal onset [50]. A significant increase in serum IGF-1 close to the onset of puberty can induce prepubertal GnRH secretion and advance the timing of puberty [51]. IGF-binding protein 3 (IGFBP-3) mainly transports IGFs in the plasma. Lower IGFBP-3 levels shorten the half-life of IGF-1 in the circulation, resulting in lower IGF-1 levels [52]. VDR acts as a transcription factor for the *IGFBP-3* gene and modulates the expression of IGFBP-3 mRNA [52, 53], which indicates that VDR influences the IGF-1 level by regulating IGFBP-3 expression. Taken together, these findings indicate that the VDR rs731236 polymorphisms that modulate IGFBP-3 mRNA expression reduce the IGF-1 level, and therefore exhibit a protective effect on earlier pubertal onset.

This case-control study investigated the association of selected SNPs with CPP risk and hormonal levels. However, there are several limitations to our study. First, our study lacks measurement on hormonal levels for control group. Therefore, for SNPs associated with both CPP risk and hormonal levels within CPP cases, the results are not enough for addressing whether or not the association with CPP is caused by its association with hormonal levels. Second, the number of loci investigated was not sufficient to fully reveal the relationship between genetic factors and CPP susceptibility. To confirm our findings, further genetic studies with more susceptibility variants and better design are required.

## 5. Conclusion

We identified associations between the gene polymorphisms rs10159082, rs7538038, rs5780218, rs3758391, rs7895833, rs3740051, and rs1544410 in KISS1, SIRT1, and VDR and CPP risk. This is the first study to report the effect of SIRT1 polymorphisms on CPP risk in Chinese girls. Further, this study was the first to report that rs1544410, rs7975232, and rs731236 in VDR affect hormonal levels in Chinese girls with CPP. In particular, rs1544410 in VDR was associated with both CPP risk and GnRH-stimulated peak FSH levels. This study provides potential genetic biomarkers for assessing CPP risk in Chinese girls. As several of the SNPs identified in our study have not previously been reported, they need to be validated in a large-scale study.

## Figures and Tables

**Table 1:** Clinical characteristics and results of hormone assays in the study population.

Characteristic	CPP (*n* = 247)	Control (*n* = 243)	*P*
Age (year)	7.71 ± 0.78	7.50 ± 1.20	0.025
Height (cm)	129.98 ± 7.08	123.28 ± 7.89	<0.001
Weight (kg)	28.32 ± 5.76	23.37 ± 4.18	<0.001
BMI (kg/m^2^)	16.63 ± 2.35	15.29 ± 1.60	<0.001
Height, SDS	1.09 ± 1.04	0.02 ± 0.88	<0.001
Weight, SDS	0.95 ± 1.00	0.06 ± 0.65	<0.001
BMI, SDS	0.71 ± 1.10	−0.03 ± 0.98	<0.001
BA (year)	9.47 ± 1.15	—	—
BA−CA (year)	1.77 ± 0.83	—	—
Peak LH (IU/L)	15.44 ± 13.07	—	—
Peak FSH (IU/L)	10.48 ± 3.79	—	—
Peak LH/FSH ratio	1.43 ± 0.92	—	—
Basal FSH (IU/L)	3.08 ± 1.50	—	—
Basal LH (IU/L)	1.02 ± 1.44	—	—
E2 (pmol/L)	101.83 ± 74.85	—	—
IGF-1 (ng/mL)	288.06 ± 96.57	—	—
IGF-1, SDS	0.79 ± 1.32	—	—

CPP: central precocious puberty; BMI: body mass index; BA: bone age; BA−CA: difference between bone age and chronological age; LH: luteinizing hormone; FSH: follicle-stimulating hormone; E2: estradiol; and IGF-1: insulin-like growth factor. Data are the mean ± standard deviation.

**Table 2:** Primary information of selected single nucleotide polymorphisms.

SNP	Chromosome	Position	Gene	Minor/major allele	Genotypes	HWE
rs10159082	chr1	204191322	kISS1	C/A	32/111/100	0.890
rs7538038	chr1	204191898	kISS1	G/A	26/109/108	1.000
rs5780218	chr1	204196482	kISS1	delA/A	35/126/82	0.286
rs7759938	chr6	104931079	LIN28B	C/T	24/95/124	0.356
rs314280	chr6	104952962	LIN28B	A/*G*	24/95/124	0.356
rs364663	chr6	104995314	LIN28B	T/A	25/96/122	0.361
rs221634	chr6	105080213	LIN28B	A/T	57/113/73	0.305
rs2234693	chr6	151842200	ER*α*	C/T	42/118/83	1.000
rs4452860	chr9	106163108	TMEM38B	G/A	51/112/80	0.301
rs7861820	chr9	106174393	TMEM38B	T/C	11/95/137	0.379
rs7895833	chr10	67863299	SIRT1	A/*G*	18/98/127	1.000
rs3758391	chr10	67883584	SIRT1	C/T	5/61/177	1.000
rs3740051	chr10	67884201	SIRT1	G/A	19/95/129	0.750
rs33957861	chr10	67887218	SIRT1	T/C	5/53/185	0.570
rs731236	chr12	47844974	VDR	G/A	0/23/220	1.000
rs7975232	chr12	47845054	VDR	A/C	21/100/122	1.000
rs1544410	chr12	47846052	VDR	T/C	0/26/217	1.000
rs2228570	chr12	47879112	VDR	A/*G*	49/125/69	0.608
rs1256049	chr14	64257333	ER*β*	T/C	28/109/106	1.000
rs3761170	chr20	8737066	PLCB1	A/*G*	3/48/192	1.000

HWE: Hardy–Weinberg equilibrium, which was estimated for each SNP using the *χ*^2^ test using individuals in the control group.

**Table 3:** Association analysis between the selected single nucleotide polymorphisms and central precocious puberty risk.

Gene	SNP	Minor/major allele	MAF		OR	95% CI	*P*
			Case	Control			
KISS1	rs10159082	C/A	0.462	0.360	1.524	1.176–1.974	**0.001**
KISS1	rs7538038	G/A	0.423	0.331	1.507	1.152–1.970	**0.003**
KISS1	rs5780218	delA/A	0.486	0.403	1.409	1.089–1.824	**0.009**
LIN28B	rs7759938	C/T	0.249	0.294	0.812	0.620–1.063	0.130
LIN28B	rs314280	A/*G*	0.261	0.294	0.861	0.659–1.124	0.271
LIN28B	rs364663	T/A	0.265	0.300	0.854	0.654–1.114	0.243
LIN28B	rs221634	A/T	0.431	0.467	0.867	0.675–1.113	0.263
ER*α*	rs2234693	C/T	0.379	0.416	0.850	0.654–1.105	0.225
TMEM38 B	rs4452860	G/A	0.498	0.440	1.242	0.973–1.584	0.082
TMEM38 B	rs7861820	T/C	0.213	0.241	0.848	0.626–1.149	0.286
SIRT1	rs7895833	A/*G*	0.338	0.276	1.348	1.023–1.777	**0.034**
SIRT1	rs3758391	C/T	0.227	0.146	1.737	1.242–2.430	**0.001**
SIRT1	rs3740051	G/A	0.207	0.274	0.689	0.511–0.928	**0.014**
SIRT1	rs33957861	T/C	0.113	0.130	0.855	0.580–1.262	0.431
VDR	rs731236	G/A	0.030	0.047	0.618	0.315–1.216	0.164
VDR	rs7975232	A/C	0.283	0.292	0.958	0.726–1.264	0.761
VDR	rs1544410	T/C	0.026	0.054	0.464	0.232–0.925	**0.029**
VDR	rs2228570	A/*G*	0.492	0.459	1.157	0.889–1.505	0.278
ER*β*	rs1256049	T/C	0.346	0.340	1.030	0.791–1.340	0.827
PLCB1	rs3761170	A/*G*	0.089	0.111	0.782	0.514–1.190	0.252

SNP: single nucleotide polymorphisms; MAF: minor allele frequency; CI: confidence interval; OR: odds ratio. *P* value was obtained from the additive model. Significant *P* values (<0.05) are highlighted in bold.

**Table 4:** Haplotype analysis in the study population.

Gene	SNP	Haplotype	Freq.	Distribution freq.		Risk	*P*
				Case	Control		
VDR	rs731236-rs7975232-rs1544410	ACC	0.712	0.717	0.708	+	0.762
		AAC	0.241	0.249	0.232	+	0.546
		GAT	0.032	0.022	0.041	−	0.091

SIRT1	rs7895833-rs3758391-rs3740051-rs33957861	ATAT	0.116	0.108	0.124	−	0.443
		GTGC	0.240	0.206	0.274	−	**0.014**
		ACAC	0.184	0.226	0.141	+	**<0.001**
		GTAC	0.445	0.449	0.440	+	0.776

KISS1	rs10159082-rs7538038-rs5780218	CG delA	0.339	0.383	0.293	+	**0.003**
		AG delA	0.013	0.007	0.019	−	0.090
		CA delA	0.034	0.040	0.029	+	0.379
		AA delA	0.059	0.056	0.062	−	0.711
		CGA	0.025	0.031	0.019	+	0.229
		CAA	0.013	0.008	0.019	−	0.137
		AAA	0.516	0.473	0.559	−	**0.007**

*P* value was obtained from the *χ*^2^ test via Haploview. Significant *P* values (<0.05) are highlighted in bold. ^+^: increased risk; ^−^: decreased risk.

**Table 5:** Association between hormonal levels and the selected single nucleotide polymorphisms.

Gene	SNP	Peak LH		Peak FSH		LH/FSH		Basal LH		Basal FSH		E2		IGF-1	
		*β*	*P*	*β*	*P*	*β*	*P*	*β*	*P*	*β*	*P*	*β*	*P*	*β*	*P*
KISS1	rs10159082	−0.988	0.410	0.261	0.457	−0.149	0.071	−0.172	0.187	0.058	0.674	−4.855	0.479	11.910	0.159
	rs7538038	−1.417	0.262	0.025	0.945	−0.145	0.095	−0.231	0.090	−0.027	0.852	−2.160	0.763	4.847	0.584
	rs5780218	0.101	0.932	0.183	0.593	−0.052	0.520	−0.062	0.628	0.113	0.397	3.550	0.595	10.010	0.225

LIN28 B	rs7759938	0.655	0.610	0.179	0.633	−0.013	0.886	−0.064	0.648	−0.001	0.996	−1.372	0.852	−3.491	0.702
	rs314280	1.386	0.271	0.104	0.779	0.073	0.398	−0.079	0.565	−0.036	0.803	−2.731	0.706	−1.962	0.827
	rs364663	0.922	0.466	0.093	0.801	0.038	0.667	−0.069	0.619	0.003	0.983	−1.281	0.860	−1.755	0.845
	rs221634	−0.978	0.418	0.001	0.997	−0.075	0.366	0.094	0.476	0.187	0.174	10.000	0.147	8.254	0.334

ER*α*	rs2234693	0.273	0.831	0.194	0.603	0.012	0.893	0.188	0.175	0.192	0.187	5.888	0.420	−6.887	0.445

TMEM38 B	rs4452860	−1.790	0.113	−0.419	0.205	−0.075	0.334	−0.213	0.084	−0.069	0.592	−2.845	0.661	8.478	0.292
	rs7861820	0.228	0.873	0.213	0.609	−0.030	0.764	−0.031	0.842	−0.043	0.794	−6.923	0.397	−19.110	0.061

SIRT1	rs7895833	−0.534	0.671	−0.148	0.687	−0.017	0.848	0.038	0.784	0.058	0.687	−2.556	0.723	−10.830	0.224
	rs3758391	−0.385	0.789	−0.166	0.693	0.023	0.818	0.098	0.533	0.065	0.693	−3.398	0.680	−14.980	0.141
	rs3740051	1.443	0.329	0.076	0.861	0.099	0.333	0.247	0.125	−0.151	0.371	−3.442	0.685	7.141	0.496
	rs33957861	−0.950	0.625	−0.329	0.563	−0.040	0.764	−0.130	0.540	0.038	0.864	−0.270	0.981	−3.310	0.810

VDR	rs731236	−4.516	0.194	−1.029	0.311	−0.227	0.345	−0.677	0.075	−0.653	0.101	−25.780	0.197	−50.360	**0.041**
	rs7975232	−1.766	0.185	−0.535	0.169	−0.096	0.294	−0.262	0.070	−-0.391	**0.010**	−12.190	0.109	−7.230	0.444
	rs1544410	−6.135	0.100	−2.181	**0.045**	−0.268	0.299	−0.655	0.109	−0.569	0.185	−18.240	0.396	−28.330	0.286
	rs2228570	1.581	0.214	0.195	0.600	0.111	0.206	0.016	0.906	0.094	0.521	5.049	0.490	3.902	0.666

ER*β*	rs1256049	1.683	0.174	0.683	0.058	0.066	0.443	0.131	0.333	0.012	0.934	−5.937	0.402	−3.799	0.666

PLCB1	rs3761170	−0.995	0.630	0.270	0.654	−0.110	0.440	−0.021	0.925	0.151	0.524	1.739	0.883	−7.210	0.622

SNP: single nucleotide polymorphisms; LH: luteinizing hormone; FSH: follicle-stimulating hormone; E2: estradiol; IGF-1: insulin-1ike growth factor. Linear regression under the additive model was used to assess the associations between SNPs and peak LH, peak FSH, peak LH/FSH ratio, basal LH, basal FSH, E2, and IGF-1, adjusting for age and body mass index in CPP groups. Significant *P* values (<0.05) are highlighted in bold.

## Data Availability

The data used to support the findings of this study are available from the corresponding author upon reasonable request.

## Supplementary Materials

Supplementary Materials Table S1. Primers details. STROBE-checklist-v4-combined.
