# Supplementary material for: Gene Polymorphisms Associated with Central Precocious Puberty and Hormone Levels in Chinese Girls
Source: Int J Endocrinol. 2022 Aug 21;2022:9450663. doi: 10.1155/2022/9450663 (PMC9420594; doi:10.1155/2022/9450663)
Supplement: Supplementary Materials — Table S1. Primers details. STROBE-checklist-v4-combined. [file 9450663.f1.zip › Supplementary Material.docx]

# International Journal of Endocrinology

**Supplementary Information**

**Gene polymorphisms associated with central precocious puberty and hormone levels in Chinese girls**

Yunwei Li^†,1,2,3^, Na Tao^†,4^, Minghui Chen^2^, Jiang Chu^2^, Xinwei Huang^5^, Xiangyang Kong^2^

^1^Faculty of Life Science and Biotechnology, Kunming University of Science and Technology, Kunming City 650500, Yunnan Province, China

^2^Medical School, Kunming University of Science and Technology, Kunming City 650500, Yunnan Province, China

^3^Department of Pharmacy, Kunming Children’s Hospital, Kunming City 650228, Yunnan Province, China

^4^Department of Endocrinology, Genetics and Metabolism of Children, Kunming Children’s Hospital, Kunming City 650228, Yunnan Province, China

^5^Translational Research Institute of Brain and Brain-Like Intelligence, Shanghai Fourth People's Hospital, School of Medicine, Tongji University, Shanghai 200434, China.

^†^ Yunwei Li and Na Tao contributed equally to this work and share first authorship.

*Correspondence should be addressed to

Xinwei Huang

[huanggenetics@tongji.edu.cn](mailto:huanggenetics@tongji.edu.cn)

ORCID: 0000-0002-8568-6261

Xiangyang Kong

[kxy2772@yahoo.com](mailto:kxy2772@yahoo.com)

**Table S1** Primers details

| SNP | Polymorphism | Forward primer (5`-3`) | Reverse primer (5`-3`) |
| --- | --- | --- | --- |
| rs3758391 | C/T | CAGATGCCATAACAAACACT | CCCTTCCACTTTCCTCTC |
| rs7895833 | A/G | TGGTTCATTTCACATGGTAAT | TAGACAGGGCAGGATAAC |
| rs3740051 | A/G | GTGAGGAGAGTGGGAAAG | CTGAAGCTGGCTAAGACT |
| rs33957861 | C/T | TGGAATTAGAGGCGTGAG | ATTGGAACAGTTTCTTCATCTT |
| rs4452860 | A/G | ATTATGAACAAAGGAATGGAGAT | GAGGGAATGTGGAGAGATT |
| rs10159082 | A/C | GTAGTAACAGAAATCGAGAAAGT | TTGAGTCCTTATGTAGCTTATTG |
| rs7538038 | A/G | ACCTCACCTAAAGCCTTT | GTCTTCTGTCCTGGGATC |
| rs5780218 | AA/A | GGTGAATGTCCAGAGGGT | GTCTGAGGAGGAGGGAGG |
| rs2234693 | C/T | TGTTGTCCATCAGTTCATCT | TCAGAACCATTAGAGACCAAT |
| rs1256049 | C/T | TAACACCTCCATCCAACAG | TCTCTACACACACAGGGA |
| rs7975232 | A/C | TTCTGGATCATCTTGGCATA | CGGTCAGCAGTCATAGAG |
| rs1544410 | C/T | GAATGTTGAGCCCAGTTC | ACTAGATAAGCAGGGTTCC |
| rs731236 | A/G | TTCTGGATCATCTTGGCATA | CGGTCAGCAGTCATAGAG |
| rs2228570 | A/G | GTCAAAGTCTCCAGGGTC | CACTGACTCTGGCTCTGA |
| rs7759938 | C/T | GACCAAATGTCCTAACACTTT | GCTTTATGGGAGGGAGAG |
| rs314280 | A/G | GTGTGAAGCCAGAGCATT | TTCTTGACATTCCTACGACAT |
| rs221634 | A/T | TGGTTCTCGTTAGAGTGAT | GTACCTTCTTGCAGATGTT |
| rs364663 | A/T | TTGGTAGACTATTGGTTGTAGA | TCACTATAATTCTCATGCTTCTG |
| rs3761170 | A/G | AATTCCTTATGGATTGATTGATT | AATGCCTTCCTCCTTGT |
| rs7861820 | C/T | AATTGGGAGGGAGACAG | GCAGATAAGGTACATACTATTC |
